# Association Between Pre-pregnancy Obesity and Adverse Obstetric Outcomes: A Retrospective Cohort Study at a Secondary Care Center in Tbilisi, Georgia

**DOI:** 10.7759/cureus.116026

**Published:** 2026-09-09

**Authors:** Niko Koridze, Giorgi Mumladze, Mamuka Nemsadze, Mariam Mamukashvili, Ioseb Nozadze, Vakhtang Mazanashvili, Mariami Varazashvili, Tsotne Dadiani, Mariam Morchadze, Keti Kebadze

**Affiliations:** 1 Obstetrics and Gynaecology, Zhordania Medical Center, Tbilisi, GEO; 2 Obstetrics and Gynaecology, Caucasus University, Tbilisi, GEO; 3 Medicine, Tbilisi State Medical University, Tbilisi, GEO; 4 Medicine, Caucasus University, Tbilisi, GEO; 5 Medicine, David Tvildiani Medical University, Tbilisi, GEO

**Keywords:** cesarean delivery, hypertensive disorders, macrosomia, maternal obesity, obesity, postpartum hemorrhage, pregnancy outcomes, pre-pregnancy bmi, preterm birth, retrospective cohort

## Abstract

Background

Maternal obesity is an increasingly important public health concern and has been consistently associated with adverse pregnancy outcomes, including hypertensive disorders of pregnancy, cesarean delivery, fetal overgrowth, and preterm birth (PTB). Local institutional data remain valuable because patient characteristics and obstetric practice patterns may vary across healthcare settings. This study aimed to evaluate the unadjusted and adjusted associations between pre-pregnancy obesity and selected adverse obstetric outcomes - hypertensive disorders of pregnancy, postpartum hemorrhage (PPH), cesarean delivery, macrosomia, and PTB - among women delivering at Zhordania Medical Center, a secondary care center in Tbilisi, Georgia.

Methods

This retrospective cohort study included 428 pregnant women with documented pre-pregnancy body mass index (BMI) who delivered at Zhordania Medical Center during the study period. Participants were divided into two groups according to pre-pregnancy BMI: BMI ≥ 30 kg/m² (obese group, n = 145) and BMI < 30 kg/m² (non-obese group, n = 283). The outcomes of interest were hypertensive disorders of pregnancy, PPH, cesarean delivery, macrosomia, and PTB. Categorical variables were summarized as frequencies and percentages, and group comparisons were performed using the chi-squared test or Fisher's exact test, as appropriate. Odds ratios (ORs) with 95% confidence intervals (CIs) were calculated using univariable logistic regression; multivariable logistic regression, adjusting for maternal age, parity, and (where estimable) prior cesarean delivery, was subsequently performed using patient-level data. A two-sided p-value < 0.05 was considered statistically significant.

Results

Women with pre-pregnancy BMI ≥ 30 kg/m² had significantly higher odds of hypertensive disorders of pregnancy than women with BMI < 30 kg/m² (26/145 vs. 14/283; OR 4.20, 95% CI 2.12-8.32; p < 0.001; adjusted OR 3.80, 95% CI 1.88-7.69). Cesarean delivery was also more common in the obese group (85/145 vs. 126/283; OR 1.77, 95% CI 1.18-2.65; p = 0.006; adjusted OR 1.83, 95% CI 1.19-2.81). Macrosomia, defined as birth weight > 4,000 g, occurred more frequently among women with obesity (23/145 vs. 23/283; OR 2.13, 95% CI 1.15-3.95; p = 0.014; adjusted OR 2.02, 95% CI 1.07-3.82). No statistically significant associations were observed for PPH (9/145 vs. 11/283; OR 1.64, 95% CI 0.66-4.04; p = 0.28) or PTB (15/145 vs. 19/283; OR 1.60, 95% CI 0.79-3.26; p = 0.19), though the achieved sample provided limited power (approximately 21% and 27%, respectively) to detect effects of this magnitude.

Conclusions

Pre-pregnancy obesity was associated with increased odds of hypertensive disorders of pregnancy, cesarean delivery, and macrosomia in this retrospective cohort from a secondary care center in Georgia, independent of maternal age, parity, and prior cesarean delivery where adjustment was feasible. These associative findings highlight the importance of preconception counseling and enhanced antenatal surveillance for women with obesity, although causal inference requires confirmation in larger, prospective studies.

## Introduction

Obesity among women of reproductive age has become a major public health issue worldwide and is increasingly encountered in obstetric practice [1,2]. Global estimates indicate that approximately 16% of adults were living with obesity in 2022, with prevalence having more than doubled since 1990, and women of reproductive age are disproportionately affected in many regions [3]. Maternal obesity has been associated with a broad spectrum of adverse maternal and neonatal outcomes, including hypertensive disorders of pregnancy, gestational diabetes, cesarean delivery, fetal overgrowth, postpartum complications, and medically indicated preterm birth [1,2,4,5].

The pathophysiological relationship between maternal obesity and pregnancy complications is multifactorial. Obesity is associated with chronic low-grade inflammation, endothelial dysfunction, oxidative stress, altered placentation, and insulin resistance, all of which may adversely affect maternal vascular adaptation and fetal growth [2,6]. A recent dose-response meta-analysis pooling data from cohorts comprising over 50 million pregnancies found that pre-pregnancy obesity was associated with a substantially increased risk of hypertensive disorders of pregnancy relative to normal-weight women, with risk rising progressively across the body mass index (BMI) range [7]. In clinical practice, obesity may also complicate intrapartum management by increasing technical difficulty during fetal monitoring and operative delivery [1].

Prior literature has shown that the risk of obstetric complications tends to rise with increasing BMI class [2]. Large cohort data summarized by the American Society for Reproductive Medicine have shown higher rates of gestational hypertensive disorders, cesarean delivery, and large-for-gestational-age birth among women with obesity compared with those with normal BMI [2]. A systematic review and meta-analysis of pregnancy complications across BMI categories similarly reported that women with obesity had more than three times the odds of gestational hypertension compared with normal-weight women [8]. With respect to delivery mode, multicenter cohort data have demonstrated a stepwise increase in cesarean delivery risk with each unit increase in maternal BMI, an effect that persists after adjustment for age, parity, and labor induction status [9]. A comparable retrospective cohort study conducted at a tertiary hospital in Bulgaria similarly found a markedly higher proportion of hypertensive disorders among women with obesity than among non-obese women, reinforcing the consistency of this association across different regional settings in Eastern Europe [10].

The American College of Obstetricians and Gynecologists (ACOG) recognizes obesity in pregnancy as a risk factor for macrosomia, cesarean delivery, and preterm birth related to pregnancy complications such as pre-eclampsia [1]. In addition, ACOG Practice Bulletin No. 173 provides guidance on the management of fetal macrosomia and highlights maternal obesity as an important risk factor for excessive fetal growth [11].

In Georgia, national data indicate that 26.8% of adult women are living with obesity, a prevalence considerably higher than the Western Asia regional average of 10.3% for women [12], underscoring the local relevance of obesity as a risk factor for adverse obstetric outcomes in this population. Georgia also has one of the higher national cesarean delivery rates internationally, with nationwide birth registry data reporting rates of approximately 40%-45% [13,14], a context relevant to the interpretation of delivery-mode findings in the present study.

Despite extensive international literature, local data remain important because obstetric practice patterns, referral pathways, and patient characteristics differ by country and institution. Evidence from Georgia remains limited. Therefore, this retrospective cohort study aimed to assess the unadjusted and, where feasible, multivariable-adjusted associations between pre-pregnancy obesity and five pre-specified obstetric outcomes of interest - hypertensive disorders of pregnancy, postpartum hemorrhage, cesarean delivery, macrosomia, and preterm birth - among women delivering at Zhordania Medical Center, a secondary care center in Tbilisi, Georgia.

## Materials and methods

Study design and setting

This was a retrospective cohort study conducted at Zhordania Medical Center, a secondary care center in Tbilisi, Georgia. Medical records of women who delivered at the institution between July 31, 2024, and July 30, 2025, were reviewed retrospectively.

Study population

A total of 428 pregnant women with documented pre-pregnancy BMI and available delivery outcome data were included in the analysis. Participants were categorized into two groups according to pre-pregnancy BMI: an obese group, defined as BMI ≥ 30 kg/m² (n = 145), and a non-obese group, defined as BMI < 30 kg/m² (n = 283). Pre-pregnancy BMI was calculated from self-reported pre-pregnancy weight, recorded by the attending obstetrician at the first antenatal visit, together with measured height.

Sample size justification

This study did not use a pre-specified, calculated sample size. Instead, the cohort comprised a consecutive convenience sample consisting of all women who delivered at Zhordania Medical Center during the defined study period and met the inclusion criteria below. This approach is common in single-center retrospective chart-review studies, where the available denominator is determined by institutional delivery volume over the observation window rather than by an a priori power calculation. As a post hoc reference, the achieved sample of 145 women with obesity and 283 women without obesity provided more than 80% power to detect odds ratios (ORs) of approximately 2.5 or greater for outcomes with a baseline prevalence near 10% in the non-obese group, using a two-sided alpha of 0.05. For postpartum hemorrhage (observed prevalence 3.9% in the non-obese group), the achieved sample had approximately 21% power to detect the observed OR of 1.64; for preterm birth (observed prevalence 6.7%), power was approximately 27% to detect the observed OR of 1.60. These findings indicate that the study was underpowered to detect effects of this magnitude, which likely explains the non-significant results for these two outcomes.

Inclusion and exclusion criteria

Women were eligible if they delivered at Zhordania Medical Center during the study period and had available pre-pregnancy BMI documentation and obstetric outcome data. Women were excluded if they had multiple gestation, a pregnancy conceived via in vitro fertilization (IVF), a major fetal anomaly, an incomplete medical chart, a miscarriage before viability, a delivery that occurred at another institution, or missing BMI documentation.

Exposure and outcomes

The five outcomes described below were pre-specified a priori as co-equal outcomes of interest, reflecting the study's broad aim of characterizing the obstetric burden associated with pre-pregnancy obesity at this institution, rather than a single primary hypothesis with secondary outcomes. The primary exposure was pre-pregnancy obesity, defined as BMI ≥ 30 kg/m². The outcomes assessed were hypertensive disorders of pregnancy (including chronic hypertension, gestational hypertension, and pre-eclampsia with or without severe features), postpartum hemorrhage, cesarean section, macrosomia (defined as birth weight > 4,000 g), and preterm birth (defined as delivery before 37 completed weeks of gestation).

Statistical analysis

Statistical analysis was performed using IBM SPSS Statistics version 29 (IBM Corp., Armonk, NY, USA). Continuous variables were assessed for normality using the Shapiro-Wilk test and visual inspection of histograms and Q-Q plots. Normally distributed continuous variables were presented as mean ± standard deviation (SD), whereas non-normally distributed variables were summarized as median and interquartile range (IQR). Categorical variables were presented as frequency and percentage.

Comparisons between groups were performed using Student's t-test for normally distributed continuous variables and the Mann-Whitney U test for non-normally distributed continuous variables. The chi-squared test was used for categorical comparisons in which all expected cell counts were 5 or greater; Fisher's exact test was used instead when this assumption was not met.

Univariable logistic regression was used to estimate the association between pre-pregnancy obesity and each adverse obstetric outcome. Results were reported as ORs with 95% confidence intervals (CIs). Following access to patient-level data, multivariable logistic regression was subsequently performed for outcomes showing a significant univariable association, adjusting for maternal age, parity, and prior cesarean delivery. For the cesarean delivery outcome, prior cesarean delivery was excluded from the multivariable model owing to complete separation: all women with a prior cesarean delivery in this cohort underwent repeat cesarean delivery, precluding stable estimation of a coefficient for this covariate. Chronic hypertension was not included as a covariate given the small number of affected women. A two-sided p-value < 0.05 was considered statistically significant.

Ethical approval

This retrospective study utilized de-identified patient data. According to the policies of our institution, formal Institutional Review Board (IRB)/Ethics Committee approval was not required for retrospective research involving fully de-identified data. The study was conducted in accordance with the ethical principles of the Declaration of Helsinki.

## Results

A total of 428 women were included in the study, of whom 145 (33.9%) were in the obese group and 283 (66.1%) were in the non-obese group. The baseline demographic and clinical characteristics of the two groups are presented in Table 1. Mean maternal age was similar between groups (33.0 ± 5.1 years in the obese group vs. 32.1 ± 5.3 years in the non-obese group; t = 1.78, df = 426, p = 0.075). Women in the obese group had significantly higher parity than women in the non-obese group (median 2, IQR 1-3, vs. median 1, IQR 1-2, respectively; Mann-Whitney U = 23,605.5, p = 0.001). The proportion of women with a prior cesarean delivery was 18.6% in the obese group and 13.8% in the non-obese group, a difference that was not statistically significant (χ² = 1.72, df = 1, p = 0.19).

**Table 1:** Baseline demographic and clinical characteristics of the study population, by pre-pregnancy BMI group Maternal age compared using the independent-samples t-test; parity compared using the Mann-Whitney U test; prior cesarean delivery compared using the chi-squared test. Parity data were available for 142 of 145 obese women and 282 of 283 non-obese women due to incomplete documentation in a small number of source charts. A p-value < 0.05 was considered statistically significant. BMI: body mass index; SD: standard deviation; IQR: interquartile range

Characteristic	Obese group (BMI ≥ 30 kg/m²) (n = 145)	Non-obese group (BMI < 30 kg/m²) (n = 283)	p-value
Maternal age (years), mean ± SD	33.0 ± 5.1	32.1 ± 5.3	0.075
Parity, median (IQR)	2 (1-3)	1 (1-2)	0.001
Prior cesarean delivery, n (%)	27 (18.6)	39 (13.8)	0.19

Hypertensive disorders of pregnancy occurred in 26 of 145 women (17.9%) in the obese group and 14 of 283 women (4.9%) in the non-obese group. Women with pre-pregnancy BMI ≥ 30 kg/m² had significantly higher odds of hypertensive disorders of pregnancy than women with BMI < 30 kg/m² (OR 4.20, 95% CI 2.12-8.32; χ² = 19.08, df = 1, p < 0.001), an association that remained significant after multivariable adjustment for maternal age, parity, and prior cesarean delivery (adjusted OR 3.80, 95% CI 1.88-7.69, p < 0.001), as shown in Tables 2, 3.

**Table 2:** Obstetric outcomes according to pre-pregnancy BMI group (univariable analysis) Non-obese group (BMI < 30 kg/m²) is the reference category. A p-value < 0.05 was considered statistically significant. BMI: body mass index; OR: odds ratio; CI: confidence interval

Outcome	BMI ≥ 30 kg/m² (n = 145), n (%)	BMI < 30 kg/m² (n = 283), n (%)	OR (95% CI)	p-value
Hypertensive disorders of pregnancy	26 (17.9)	14 (4.9)	4.20 (2.12-8.32)	<0.001
Postpartum hemorrhage	9 (6.2)	11 (3.9)	1.64 (0.66-4.04)	0.28
Cesarean section	85 (58.6)	126 (44.5)	1.77 (1.18-2.65)	0.006
Macrosomia (>4,000 g)	23 (15.9)	23 (8.1)	2.13 (1.15-3.95)	0.014
Preterm birth	15 (10.3)	19 (6.7)	1.60 (0.79-3.26)	0.19

**Table 3:** Multivariable-adjusted associations between pre-pregnancy obesity and significant obstetric outcomes Adjusted for the covariates listed via multivariable logistic regression. Prior cesarean delivery was excluded from the cesarean-section model due to complete separation (all women with a prior cesarean delivery underwent repeat cesarean delivery in this cohort). Chronic hypertension was not included as a covariate given the small number of affected women. OR: odds ratio; CI: confidence interval

Outcome	Adjusted OR (95% CI)	p-value	Covariates in model
Hypertensive disorders of pregnancy	3.80 (1.88-7.69)	<0.001	Age, parity, prior cesarean
Cesarean section	1.83 (1.19-2.81)	0.006	Age, parity
Macrosomia (>4,000 g)	2.02 (1.07-3.82)	0.030	Age, parity, prior cesarean

Postpartum hemorrhage occurred in nine of 145 women (6.2%) in the obese group and 11 of 283 women (3.9%) in the non-obese group. The point estimate suggested an approximately 1.6-fold increase in the odds of postpartum hemorrhage among women with obesity; however, the 95% CI was wide and crossed unity (OR 1.64, 95% CI 0.66-4.04; p = 0.28), and this association did not reach statistical significance. Given the modest observed power for this outcome (approximately 21%), this result should not be interpreted as evidence of no association, but rather as inconclusive given the sample size available.

Cesarean section was performed in 85 of 145 women (58.6%) in the obese group and 126 of 283 women (44.5%) in the non-obese group. Pre-pregnancy obesity was associated with significantly higher odds of cesarean delivery (OR 1.77, 95% CI 1.18-2.65; χ² = 7.62, df = 1, p = 0.006), an association that persisted after multivariable adjustment for maternal age and parity (adjusted OR 1.83, 95% CI 1.19-2.81, p = 0.006; prior cesarean delivery was excluded from this model due to complete separation, as detailed in Statistical analysis).

Macrosomia, defined as birth weight > 4,000 g, occurred in 23 of 145 neonates (15.9%) in the obese group and 23 of 283 neonates (8.1%) in the non-obese group. Women with pre-pregnancy obesity had significantly higher odds of delivering a macrosomic neonate (OR 2.13, 95% CI 1.15-3.95; χ² = 5.98, df = 1, p = 0.014), an association that remained significant after multivariable adjustment for maternal age, parity, and prior cesarean delivery (adjusted OR 2.02, 95% CI 1.07-3.82, p = 0.030).

Preterm birth occurred in 15 of 145 women (10.3%) in the obese group and 19 of 283 women (6.7%) in the non-obese group. Although preterm birth was numerically more frequent in the obese group, the association was not statistically significant (OR 1.60, 95% CI 0.79-3.26; χ² = 1.73, df = 1, p = 0.19), and the achieved sample provided limited power (approximately 27%) to detect an effect of this magnitude. Preterm birth was analyzed as a composite outcome; distinguishing between spontaneous and medically indicated preterm birth was not possible with the available data, but would be informative, as obesity is more consistently associated with medically indicated preterm birth (e.g., related to pre-eclampsia) than with spontaneous preterm labor. All five outcomes are summarized in Table 2.

## Discussion

This retrospective cohort study assessed the association between pre-pregnancy obesity and adverse obstetric outcomes at a secondary care center in Tbilisi, Georgia. The main findings were that women with pre-pregnancy BMI ≥ 30 kg/m² had significantly higher odds of hypertensive disorders of pregnancy, cesarean delivery, and macrosomia compared with women with BMI < 30 kg/m², associations that persisted after multivariable adjustment for maternal age, parity, and (where estimable) prior cesarean delivery, as summarized in Tables 2, 3. No statistically significant associations were identified for postpartum hemorrhage or preterm birth, though the study was underpowered to detect effects of the magnitude observed for these outcomes.

The observed association between pre-pregnancy obesity and hypertensive disorders of pregnancy is consistent with the broader literature. Obesity is known to increase the risk of gestational hypertensive disorders through mechanisms that include endothelial dysfunction, chronic inflammation, sympathetic activation, and altered vascular adaptation to pregnancy [6,7]. Large cohort data summarized in a major reproductive medicine committee opinion showed that the risk of gestational hypertensive disorders rises progressively across obesity classes [2]. A recent dose-response meta-analysis of cohorts encompassing over 50 million pregnancies similarly reported a substantially elevated risk of hypertensive disorders among women with pre-pregnancy obesity compared with normal-weight women [7]. A comparably elevated proportion of hypertensive disorders among obese women has also been reported in a retrospective cohort from a tertiary center in Bulgaria, lending regional plausibility to the present estimate [10]. The higher prevalence of pre-existing chronic hypertension in the obese group (2.9% vs. 0.3%) may have contributed to the elevated risk of hypertensive disorders of pregnancy observed in this cohort. In multivariable analysis adjusting for maternal age, parity, and prior cesarean delivery, the association between obesity and hypertensive disorders remained significant (adjusted OR 3.80, 95% CI 1.88-7.69). Given the small number of women with chronic hypertension (n = 5), this variable could not be reliably incorporated into the multivariable model, and the magnitude of its independent contribution cannot be precisely determined from the available data.

The cesarean delivery finding is also concordant with prior evidence. ACOG notes that women with obesity are more likely to require cesarean birth and may experience longer labors and more difficult intrapartum monitoring [1]. Multicenter cohort data have demonstrated a graded, dose-dependent relationship between increasing maternal BMI and cesarean delivery risk, with each 1 kg/m² increase in BMI associated with an incremental rise in risk independent of age, parity, and induction status [9]. Other cohort studies have reported two- to threefold increases in cesarean risk among women with obesity compared with leaner reference groups [12]. In the present cohort, 58.6% of women in the obese group underwent cesarean delivery compared with 44.5% of women in the non-obese group (OR 1.77, 95% CI 1.18-2.65). This association persisted after multivariable adjustment for maternal age and parity (adjusted OR 1.83, 95% CI 1.19-2.81, p = 0.006), and its magnitude is consistent with the two- to threefold increases reported in prior literature [12]. Prior cesarean delivery could not be included as a covariate in this model owing to complete separation: all women with a prior cesarean delivery in this cohort underwent repeat cesarean delivery, itself a notable finding suggesting a very low institutional rate of trial of labor after cesarean, which may independently contribute to the overall high cesarean rate observed at this center. The overall cesarean rates observed in this cohort, while high relative to many international series, are consistent with national data: Georgia has one of the highest national cesarean section rates internationally, reported at approximately 40%-45% in nationwide birth registry studies [14,15], reflecting broader institutional and national practice patterns rather than a feature specific to this cohort.

Macrosomia was significantly more common among women with obesity in this study. This is consistent with accepted obstetric literature identifying maternal obesity as an important risk factor for fetal overgrowth [1,5]. ACOG describes macrosomia as commonly referring to birth weight greater than 4,000 g, and maternal obesity is recognized as one of the major contributing factors [1,5]. Excess maternal adiposity is linked to insulin resistance and increased nutrient availability to the fetus, both of which may promote excessive fetal growth [5,6]. This association remained significant after multivariable adjustment (adjusted OR 2.02, 95% CI 1.07-3.82, p = 0.030).

In contrast, postpartum hemorrhage and preterm birth were not significantly associated with pre-pregnancy obesity in this cohort. Prior literature has reported inconsistent findings for these outcomes. Some studies have suggested increased risks of postpartum hemorrhage and preterm birth among women with obesity, whereas others have found that the relationship is influenced by comorbidities, obstetric intervention, and the distinction between spontaneous and medically indicated preterm birth [1,4,6]. In the present study, both outcomes were numerically more common in the obese group, but the CIs crossed 1 in both cases; the point estimates, however, are consistent in direction and approximate magnitude with previously reported associations between obesity and these outcomes, and should not be dismissed as null findings given the limited power of this cohort to detect effects of this size. As outlined in the sample size justification above, the achieved sample provided reasonable power to detect the effects observed for hypertensive disorders and cesarean delivery, but was underpowered (approximately 21% and 27%, respectively) to detect more moderate associations of the magnitude observed for postpartum hemorrhage and preterm birth.

The present findings are clinically relevant in the Georgian setting. Even with multivariable adjustment for available confounders, the data suggest that pre-pregnancy obesity identifies a subgroup at substantially increased risk for hypertensive morbidity, cesarean delivery, and fetal macrosomia. From a practical standpoint, these results support greater attention to preconception counseling, optimization of maternal weight before pregnancy, and closer antenatal surveillance in women entering pregnancy with obesity [1,2].

Limitations

This study has several limitations. First, the retrospective single-center design limits generalizability and does not allow causal inference. Second, while we were able to perform multivariable logistic regression using patient-level data to adjust for maternal age, parity, and prior cesarean delivery (see Results), we were unable to adjust for smoking status or chronic comorbidities other than hypertension, as these were either not systematically recorded or, in the case of chronic hypertension, present in too few women for reliable inclusion in multivariable models; residual confounding from these factors cannot be excluded. Third, the study did not use a pre-calculated sample size, and the achieved sample, while adequate to detect the effects observed for hypertensive disorders and cesarean delivery, was underpowered for less frequent outcomes such as postpartum hemorrhage and preterm birth (observed post hoc power approximately 21% and 27%, respectively). Fourth, preterm birth was analyzed as a composite outcome, without distinguishing between spontaneous and medically indicated preterm birth. This distinction may be particularly important when interpreting the relationship between obesity and prematurity, as obesity has been more consistently associated with medically indicated preterm birth - for example, iatrogenic delivery for pre-eclampsia or fetal growth concerns - than with spontaneous preterm labor. The composite outcome used here may therefore understate or obscure a more specific association with medically indicated preterm birth.

Strengths

Despite these limitations, the study has several strengths. It addresses an important clinical question using real-world institutional data from Georgia, where published evidence remains limited. The cohort design, clearly defined BMI exposure groups, and reporting of ORs with CIs and corresponding test statistics improve the interpretability and clinical applicability of the findings. Additionally, by providing corrected, verified institutional estimates for hypertensive disorders, cesarean delivery, and macrosomia in relation to pre-pregnancy obesity, this study offers a baseline against which future, larger, and ideally multicenter studies in Georgia can be compared.

## Conclusions

In this retrospective cohort study conducted at a secondary care center in Tbilisi, Georgia, pre-pregnancy obesity was significantly associated with hypertensive disorders of pregnancy, cesarean section, and macrosomia, defined as birth weight > 4,000 g, independent of maternal age, parity, and prior cesarean delivery where adjustment was feasible. No statistically significant association was observed for postpartum hemorrhage or preterm birth, though the study was likely underpowered to detect effects of the magnitude observed for these outcomes. These findings suggest an association between pre-pregnancy obesity and adverse obstetric outcomes in this Georgian cohort. They underscore the importance of preconception weight optimization and enhanced antenatal surveillance for women with obesity, although causal inference requires confirmation in larger, prospective, multicenter studies.
